# Application of Spectrometric Technologies in the Monitoring and Control of Foods and Beverages

**DOI:** 10.3390/foods10050948

**Published:** 2021-04-26

**Authors:** Ricard Boqué, Barbara Giussani

**Affiliations:** 1 Department of Analytical Chemistry and Organic Chemistry, Universitat Rovira i Virgili, 43007 Tarragona, Catalunya, Spain; 2 Dipartimento di Scienza e Alta Tecnologia, Università degli Studi dell’Insubria, Via Valleggio 11, 22100 Como, Italy

In order to obtain high-quality products and gain a competitive advantage, food producers seek improved manufacturing processes, particularly when physicochemical and sensory properties add significant value to the product. Improving the chemical and sensory properties requires a deeper understanding and control of the production process.

The present Special Issue aims to visualize the recent advances of spectrometric techniques, such as infrared and Raman spectroscopy, and mass spectrometry (ICP-MS, LC-MS and GC–MS) in the monitoring and control of foodstuffs.

Among their applications, spectroscopic techniques seem to be the most preferred: often the measurement of a spectrum can be made on the sample as it is, without any pre-treatment, which is certainly a driving force to employ such techniques. Several of the research works presented could also lay the foundations for the development of process control techniques, as well as the quality control of food and beverages.

While benchtop instrumentation still seems to be the most used, it is interesting to note the increase in applications in which portable instrumentation is employed. Mario Li Vigni and coauthors [1] used a portable Raman sensor to study grated protected denomination of origin (PDO) Parmigiano Reggiano cheese. By using the class modeling approach (Soft Independent Modeling of Class Analogy, SIMCA), they developed an authenticity model, while using a multivariate calibration model they verified the compliance of the product with respect to the production protocol. Jordi Riu and coauthors [2] used two miniaturized near-infra-red (NIR) spectrometers to predict fat and protein contents and the presence or absence of lactose in commercial milk samples. Multivariate calibration was also used in this case to perform the predictions and the classification. The results are very satisfactory and are even better when the signals of the two sensors are coupled in a data fusion approach: spectral data recorded by the two instruments were considered simultaneously since they used different wavelength ranges.

Benchtop NIR spectroscopy was applied in several interesting control quality strategies. Jong-Rak Park and coauthors [3] employed NIR spectroscopy to determine, in a non-destructive way, the piperine content in black pepper. High-performance liquid chromatography (HPLC) was used to obtain the reference value. A multivariate regression model aiming to quantify piperine from the NIR spectra was then built. Considering that an NIR measurement takes 2 min per sample, while HPLC takes 20 min for the same analysis, and considering also that the HPLC method requires a sample pretreatment and extra time for data interpretation, the authors stated that NIRS was confirmed to be the more efficient analytical method. Manuela Mancini and coauthors [4] used NIR spectroscopy to determine the main qualitative characteristics of strawberry fruit. Principal component analysis (PCA) was able to group fruits of different genotypes according to their NIR spectra. Multivariate regression was used to predict the qualitative parameters, thus avoiding the use of reference techniques, which are destructive. Pamela Galvin-Kin and coauthors [5] proposed a method for the detection of paprika adulteration based on near- and mid-infra-red (FTIR) spectroscopies. NIR and FTIR were used in conjunction with chemometrics to develop methods for the detection of spent paprika in paprika as a fraudulent bulking agent. NIR performed better than FTIR in the modeling of authentic paprika, though both techniques were not always able to detect the low-level adulterations. Finally, Najeeb Ur Rehman and coauthors [6] proposed an alternative method for the quantification of caffeine in commercially available tea samples consumed in Oman based on NIR spectroscopy. In this case, the samples were not analyzed directly, instead the tea extract was prepared and analyzed by both NIRS and HPLC to make a comparison: the authors declared that the NIR method was adequate for the purpose and had significant potential, due to the quick and reproducible results it provided.

Tea was also analyzed by Gabriella Pinto and coauthors [7], in this case with the intent to determine polyphenols and metals integrating different mass spectrometry methodologies: LC–MS/MS in multiple reaction monitoring (MRM) and inductively coupled plasma mass spectrometry (ICP-MS). Additionally, in this case, a tea infusion was used as the sample, and mineralization and liquid/liquid extraction were then performed for IPC–MS and LC–MS/MS analysis, respectively. A high number of target analytes were monitored and quantified, and the authors declared that both the targeted MRM–MS and ICP analyses returned with adequate specificity and sensitivity for their applicability to food control. Polyphenols and metals were also analyzed in Italian wines by Paola Fermo and coauthors [8]. Inductively coupled plasma optical emission spectrometry (ICP–OES) and ICP–MS were used for metal analysis, while ultra-high performance liquid chromatography (UHPLC)-Orbitrap MS technique and UHPLC coupled with a diode array detector and triple-quadrupole mass spectrometer (UHPLC–DAD-QqQ–MS) were used to determine anthocyanins and non-anthocyanins, respectively. The total phenolic content (TPC) and radical scavenging activity (RSA) were measured using spectrophotometric methods. PCA applied on the data allowed to group wines according to the geographical provenance, and within the region, a differentiation according to the botanical origin was also found.

Finally, a very interesting application that could be described as process control was proposed by Lisa Rita Magnaghi and coauthors [9]. The article deals with meat spoilage, which is of great concern in the meat industry and commerce. They propose a system, a colorimetric array, to be placed over the tray containing a sample of meat, which changes color according to the degradation process. The authors validated the system with different kinds of meat, obtaining different pathways of degradation. The changes in the sensor color were monitored through photos taken of the sensor array during the process. The collected images were then transformed into a data table and treated with PCA and multiway PCA to detect the spoilage process.

In general, the papers included in the Special Issue “Application of Spectrometric Technologies in the Monitoring and Control of Foods and Beverages” come from the twofold approach of applying analytical chemistry to food analysis. On one side there is the importance of developing analytical strategies easy-to-apply directly to the sample, avoiding sample pretreatments, wastes, the use of solvents and thus optimizing (minimizing) materials and time, and possibly without bringing the samples to the lab. On the other hand, there is the need for more sophisticated techniques able to quantify analytes at very low levels of detection, with very high precision and accuracy, and in this case, compromises are not always allowed as samples have to be pretreated and time, material, and in general resources have to be invested. Chemometrics seems to be the common way to analyze the data. Modern analytical techniques return multivariate data, and always several analytical techniques are simultaneously used to describe the samples: so why should the data not be used simultaneously? Multivariate analysis is the correct way to address this issue, and this emerges from the articles reported in this Special Issue, in which chemometrics plays a key role in achieving the desired results. We would like to thank all the authors for their contributions, and we expect the results shown in this Special Issue to be a stimulus for future developments and ideas about monitoring and controlling the quality of food and beverages.

## Data Availability

Not applicable.

## References

[1] Li Vigni M., Durante C., Michelini S., Nocetti M., Cocchi M. (2020). Preliminary Assessment of Parmigiano Reggiano Authenticity by Handheld Raman Spectroscopy. Foods.

[2] Riu J., Gorla G., Chakif D., Boqué R., Giussani B. (2020). Rapid Analysis of Milk Using Low-Cost Pocket-Size NIR Spectrometers and Multivariate Analysis. Foods.

[3] Park J., Kang H., Cho J., Moon K., Kim Y. (2020). Application of Non-Destructive Rapid Determination of Piperine in Piper nigrum L. (Black Pepper) Using NIR and Multivariate Statistical Analysis: A Promising Quality Control Tool. Foods.

[4] Mancini M., Mazzoni L., Gagliardi F., Balducci F., Duca D., Toscano G., Mezzetti B., Capocasa F. (2020). Application of the Non-Destructive NIR Technique for the Evaluation of Strawberry Fruits Quality Parameters. Foods.

[5] Galvin-King P., Haughey S., Elliott C. (2020). The Detection of Substitution Adulteration of Paprika with Spent Paprika by the Application of Molecular Spectroscopy Tools. Foods.

[6] Ur Rehman N., Al-Harrasi A., Boqué R., Mabood F., Al-Broumi M., Hussain J., Alameri S. (2020). FT-NIRS Coupled with PLS Regression as a Complement to HPLC Routine Analysis of Caffeine in Tea Samples. Foods.

[7] Pinto G., Illiano A., Carpentieri A., Spinelli M., Melchiorre C., Fontanarosa C., di Serio M., Amoresano A. (2020). Quantification of Polyphenols and Metals in Chinese Tea Infusions by Mass Spectrometry. Foods.

[8] Fermo P., Comite V., Sredojević M., Ćirić I., Gašić U., Mutić J., Baošić R., Tešić Ž. (2021). Elemental Analysis and Phenolic Profiles of Selected Italian Wines. Foods.

[9] Magnaghi L., Capone F., Zanoni C., Alberti G., Quadrelli P., Biesuz R. (2020). Colorimetric Sensor Array for Monitoring, Modelling and Comparing Spoilage Processes of Different Meat and Fish Foods. Foods.

